# “Do benzodiazepines have a future in treating acute vertigo"

**DOI:** 10.1016/j.amsu.2022.104623

**Published:** 2022-09-09

**Authors:** Syeda Shahnoor, Ume Habiba, Hussain Haider Shah

**Affiliations:** Dow University of Health Sciences, Karachi, Pakistan

## 1 Background

Vertigo, a type of dizziness, has been defined by the Hearing and Equilibrium Committee of the American Academy of Otolaryngology-Head and Neck Surgery as ‘the sensation of motion when no motion is occurring relative to earth's gravity’ [1]. It is a symptom of felt motions or sensations, an illusion of rotational movements of the subject or surroundings [2]. The Central nervous system integrates vestibular, proprioceptive, and visual information to maintain balance. Vertigo can be produced by any disruption in connected pathways or by malfunctioning of two or more systems [3]. Vertigo often coexists with nausea, vomiting, nystagmus, headache, perspiration, and ringing in the ears. Patients describe feeling like they are spinning or moving in circles [4]. The most prevalent type of dizziness is vertigo which constitutes approximately 54% of reports of dizziness in primary care [6]. A survey of the general population found a 5% 12-month vertigo prevalence and 1.4% annual incidence. Dizziness and vertigo are common ED complaints with many causes. They affect 15%–20% of individuals annually, are two to three times more common in women than men, and are among the top ten reasons neurologists are referred in emergency and office-based settings [5,7,8]. This epidemiology data underlines the importance of learning more about vertigo as a symptom in vestibular diseases to improve patient management. Dizziness and vertigo can affect a person's quality of life. In a German epidemiological study, participants with vestibular vertigo and nonvestibular dizziness reported medical consultation (70% vs. 54%), sick leave (41% vs. 15%), impairment of daily activities (40% vs. 12%), and avoidance of leaving the house (19% vs 10%) [9].

## 2 Current guidelines

The best treatment modality can be elusive because of multiple concurrent causes of vertigo, broadly classified into central and peripheral causes [5]. The treatment of vertigo can be specific/curative or symptomatic. Symptomatic treatment controls acute symptoms, while curative care targets the underlying cause of vertigo [10]. Benign Paroxysmal Positional Vertigo (BPPV), which is the most common cause of vertigo in a clinical setting, improves with head rotation maneuvers [5,11]. However, for disorders like Ménière's disease, which has no cure and is treated symptomatically, medication management is beneficial in reducing acute vertigo symptoms [12]. Today, most clinicians rely on symptom control through medicines. Furthermore, in some situations, acutely symptomatic patients (nausea and vomiting) or who continue to be symptomatic despite repositioning maneuvers benefit from short-term treatment with vestibular suppressants [13]. UpToDate, the most trustworthy clinical decision support tool at the point of care, recommends treating acute vertigo, as well as nausea and emesis, with antihistamines, benzodiazepines, and antiemetics [14].

## 3 Vestibular suppressants and their mode of action

Frequently prescribed vestibular suppressants effective in the acute phase of vertigo are antihistamines and benzodiazepines. Antihistamines used to treat vertigo have anticholinergic action and reduce motion sickness associated with acute vertigo. Gamma-aminobutyric acid (GABA) is an inhibitory neurotransmitter in the vestibular system. Benzodiazepines act centrally via GABA-A receptors to boost GABA's action in the central nervous system and reduce vertigo [15]. Because of its anxiolytic effect, benzodiazepines can also help relieve the panic and anxiety that typically accompany acute vertigo. Due to their serious side effects, they are only given in small doses and withdrawn once severe vertigo and nystagmus symptoms resolve [16].

## 4 New findings

The effectiveness of vestibular suppressants for the symptomatic management of acute vertigo, with or without a clear diagnosis, is still uncertain despite their longstanding indication for this purpose. Antihistamines and benzodiazepines were both subjected to a meta-analysis and systematic review by Hunter et al. the results of which were published in JAMA Neurology on July 18, 2022. Randomized controlled trials (RCTs) comparing antihistamines or benzodiazepines to another treatment, a placebo, or no treatment at all were included in the analysis for persons with acute vertigo that lasted less than two weeks. Efficacy results from 17 studies including 1586 people from 11 countries were reviewed. Two hours after treatment, improvements on a 10- or 100-point visual analog scale (VAS) for vertigo or dizziness were the primary outcome of interest. Vertigo improvement at one week and one month, and a decrease in nausea VAS score at 2 h, were secondary outcomes.

The analysis showed that at around 2 h post-treatment, single-dose antihistamines were 16.1 points (95% CI 7.2–25.0) more efficacious than single-dose benzodiazepines in reducing the vertigo symptom scores on a 100-point visual analogue scale (VAS). The performance of antihistamines was comparable to that of other active comparators such as ondansetron, droperidol, metoclopramide, and piracetam (mean difference = 7.4, 95% CI -1.12 to 15.8). There was no evidence to suggest that antihistamines increased the likelihood of complete symptom remission after one week and one month (relative risk, RR = 1.03, 95% CI 0.56–1.89). Authors found fairly persuasive evidence that single-dose antihistamines were superior to benzodiazepines in relieving vertigo symptoms after 2 h. Furthermore, the evidence did not support an association between benzodiazepine use with an improvement in any symptom of acute vertigo at all [17].

## 5 Future prospect

According to current guidelines, patients in the acute setting/ED who come with severe nausea and vomiting require vestibular suppressants to reduce symptoms. The study by Hunter et al. reveals that antihistamines are more efficient than benzodiazepines at managing acute symptoms. Therefore, Antihistamines should be prioritized above benzodiazepines. Further randomised trials and research are necessary to compare the efficacy of various drugs to refine the management of acute vertigo. An editorial published in JAMA Neurology concludes that antihistamines may be superior to benzodiazepines in the treatment of acute vertigo, but that proper diagnosis is superior to both. The editorial also stresses the importance of determining the specific central or peripheral differential diagnosis in patients with acute vertigo, arguing that failing to do so ignores the significance of clinically relevant literature that supports disease-specific treatment [18]. One of the most crucial objectives identified in an international survey of emergency physicians was the creation of clinical decision criteria for acute vertigo [19]. The Society for Academic Emergency Medicine began writing a clinical guideline for acute dizziness and vertigo in 2021. This guideline will give exact recommendations for diagnosing and treating first-care patients with episodic and acute vestibular disorders [18]. In a cross-sectional survey, one-fourth of 9500 acutely dizzy ED patients were misdiagnosed due to a lack of clinical criteria [20]. This survey stresses the necessity of adequate guidelines for treating acute vertigo in the ED. More research and studies are needed to increase clinicians' understanding of acute vertigo, differential diagnosis, and management of such individuals.

## Ethical approval

This paper did not involve patients; therefore no ethical approval was required.

## Sources of funding

No funding was required for this paper.

## Author contribution

Syeda Shahnoor: Conceptualization, writing – original draft, final approval and agreeing to the accuracy of the work. Ume Habiba: writing - original draft, final approval and agreeing to the accuracy of the work. Hussain Haider Shah: writing - original draft, final approval and agreeing to the accuracy of the work.

## Registration of research studies

1. Name of the registry: Not Applicable
2. Unique Identifying number or registration ID: Not Applicable
3. Hyperlink to your specific registration (must be publicly accessible and will be checked): Not Applicable

## Guarantor

Syeda Shahnoor.

Ume Habiba.

Hussain Haider Shah.

## Consent

The study was not done on patients or volunteers; therefore no written consent was required.

## Declaration of competing interest

The authors declare that there is no conflict of interest.
